# Multimodal imaging of retinal metastasis masquerading as an acute retinal necrosis

**DOI:** 10.1186/s40942-018-0149-4

**Published:** 2018-11-21

**Authors:** Fukutaro Mano, Stephen A. LoBue, Kuo-Chung Chang, Tomiya Mano

**Affiliations:** 1 Suita Tokushukai Hospital Eye Center, 21-1, Senriokanishi, Suita City, Osaka 565-0814 Japan; 2 LoBue Laser and Eye Medical Centers, Temecula, CA USA; 3 Lincoln Medical Center, Bronx, NY USA

**Keywords:** Metastasis, Retina, Acute retinal necrosis, Vitrectomy, Cisplatin, Fluorouracil, Blood–brain barrier

## Abstract

**Background:**

To report the multimodal imaging and histology of a case of metastatic esophageal cancer with vitreoretinal involvement resembling acute retinal necrosis (ARN) in a patient receiving systemic chemotherapy.

**Case presentation:**

A 69-year-old Japanese man with a history of stage 4 esophageal carcinoma, treated with three cycles of 5-fluorouracil (5-FU) and cisplatin (CDDP) chemotherapy as well as 30 sessions of radiation therapy, presented with new onset of blurry vision in the right eye (OD). Visual acuity was 20/200 OD. Fundus examination OD revealed 2+ nuclear cataract, veil-like vitreous opacity, a tractional retinal detachment, and white retinal lesions in the macula and periphery masquerading as an ARN. Due to the poor view and uncertainty regarding diagnosis, combined cataract extraction and 25 gauge pars plana vitrectomy was performed. Polymerase chain reaction and cytologic analysis were performed on the vitreous samples, which was negative for all infectious entities but positive for poorly differentiated malignant cells. The vitreous biopsy was consistent with the primary endoscopic esophageal biopsy. Ultra-wide view fundus imaging revealed multifocal white intraretinal lesions in the macula and periphery. Optical coherence tomography through these white opacities displayed hyper-reflective inner retinal lesions with no choroidal involvement, suggestive of retinal metastasis. Observation and palliative support was continued until the patient passed away 3 months after diagnosis.

**Conclusion:**

Retinal metastasis may mimic infectious syndromes such as ARN and are associated with a very poor prognosis. Outside of the retina, no further central nervous system metastasis was found. 5-FU is known to cross the blood–brain-barrier but may be inadequate in preventing retinal metastasis.

## Background

Intraocular metastases commonly arise in the uveal tract, with a majority affecting the choroid [1]. Choroidal metastases generally appear as creamy white or yellow masses. Metastases may also have multifocal or bilateral involvement [2].

Conversely, metastases to the retina are rare, occurring in less than 1% of all intraocular tumor dissemination [3]. The typical presentation of retinal metastasis includes white, yellow, or brown lesions most commonly in the inner retina. Subretinal fluid, vitreous seeds, and retinal or vitreous hemorrhage are the most common fundus findings. However, due to the rarity of retinal metastases, misdiagnosis for inflammatory or infectious retinitis has occurred in different institutions [3–5].

Acute retinal necrosis (ARN) is a rare infectious ocular condition characterized by severe occlusive vasculitis, diffuse necrotizing retinitis, and moderate-to-severe vitritis [6]. Long-term complications from ARN includes retinal detachment in up to 75% of patients, macular hole or epi-retinal membrane formation, and optic or retinal atrophy [7]. ARN is predominantly caused by the herpes family such as varicella-zoster virus, cytomegalovirus, and herpes simplex 1 and 2. Immunosuppressed individuals, such as the aging population or those receiving systemic chemotherapy, may be at an increased risk of infection with a more severe disease burden, thus it is important to diagnose and intervene early when ARN is suspected in this population.

We present a very unusual case in which a patient being actively treated for stage 4 esophageal carcinoma presented with visual symptoms and fundus findings highly suspicious of ARN which is generally characterized by vitreitis, and white colored lesions of peripheral necrotizing retinitis that often leads to secondary retinal detachment. However, rapid intervention with intravitreal biopsy and fundus imaging confirmed vitreoretinal metastases from the primary tumor site. Clinical presentation, treatment strategy, and patient outcomes are discussed in our case in order to add to the common knowledge of this exceedingly rare condition.

## Case presentation

A 69-year-old Japanese man presented to our institution with decreased vision in his right eye. He had a medical history of stage 4, poorly differentiated, esophageal cancer that had been diagnosed previously via endoscopic biopsy. Positron emission tomography–computed tomography (PET–CT) revealed multifocal increases in fluorodeoxyglucose uptake into the esophagus, lung, liver, lumbar vertebrae, and mediastinal lymph nodes. The patient was treated with three cycles of fluorouracil (5-FU) and cisplatin (CDDP) chemotherapy as well as 30 sessions of radiation therapy (60 Gy) over approximately 6 weeks, three months prior to presentation.

**Fig. 1:**
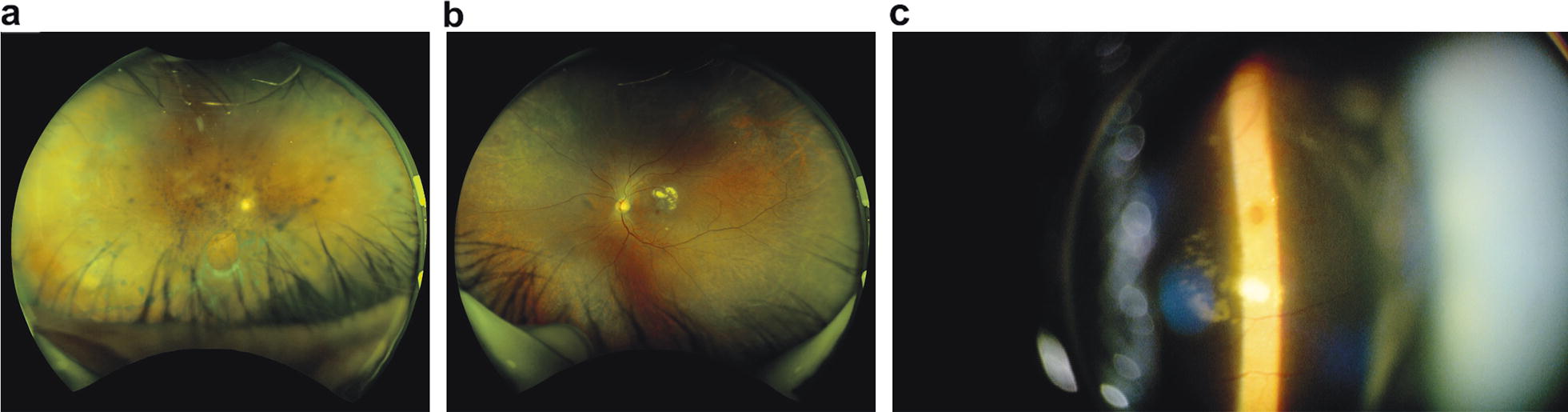
Fundus photographs obtained at presentation of a 69-year-old Japanese man with a history of stage 4 esophageal cancer. **a** In the right eye, a veil-like vitreous opacity and retinal whitening in the macular and peripheral retina were apparent. The retinal pigment epithelium appeared to be intact and no choroidal masses or lesions were visible. **b** The left eye had an old macular hole, which included hard exudates. No masses or lesions were visible. **c** Magnified fundus photograph showed a macular hole in the left eye

Visual acuity was 20/200 in the right eye (OD) and 20/600 in the left eye (OS). The patient reported previously having a macular hole in the left eye but received no surgical intervention. Anterior segment examination was normal except for 2+ nuclear cataracts in both eyes (OU). No anterior segment inflammation was present in either eye. A dilated fundus examination revealed a veil-like vitreous opacity with white retinal lesions in the macula and periphery OD, consistent with a vasculitis or possible ARN (Fig. 1a). Although the view was limited due to the thick vitreous opacity, no obvious masses were detected in the retina or choroid. Fundus examination of the left eye was normal, except for evidence of the old macular hole with hard exudates along the superior temporal arcade (Fig. 1b, c). Given the patient’s history of metastatic esophageal cancer, differential diagnoses included acute retinal necrosis (ARN), chronic uveitis, and neoplastic disease. Due to the poor view and uncertainty regarding diagnosis, surgical intervention was scheduled two days later.

A combined cataract extraction and 25 gauge pars plana vitrectomy was performed. Phacoemulsification was followed by intraocular lens implantation. Next the vitreous opacity was removed and submitted for polymerase chain reaction (PCR) testing and cytologic analyses. A peripheral tractional retinal detachment was also detected during the surgery. Therefore, a silicone oil tamponade was selected. In consideration of the possible ARN diagnosis, the patient was started on systemic antiviral (250 mg/day intravenous acyclovir for 3 days), anti-inflammatory (20 mg/day oral prednisolone for 3 days), and anti-coagulant (100 mg/day biaspirin for 3 days) therapies immediately following surgery with cooperation with the internal medicine department.

**Fig. 2:**
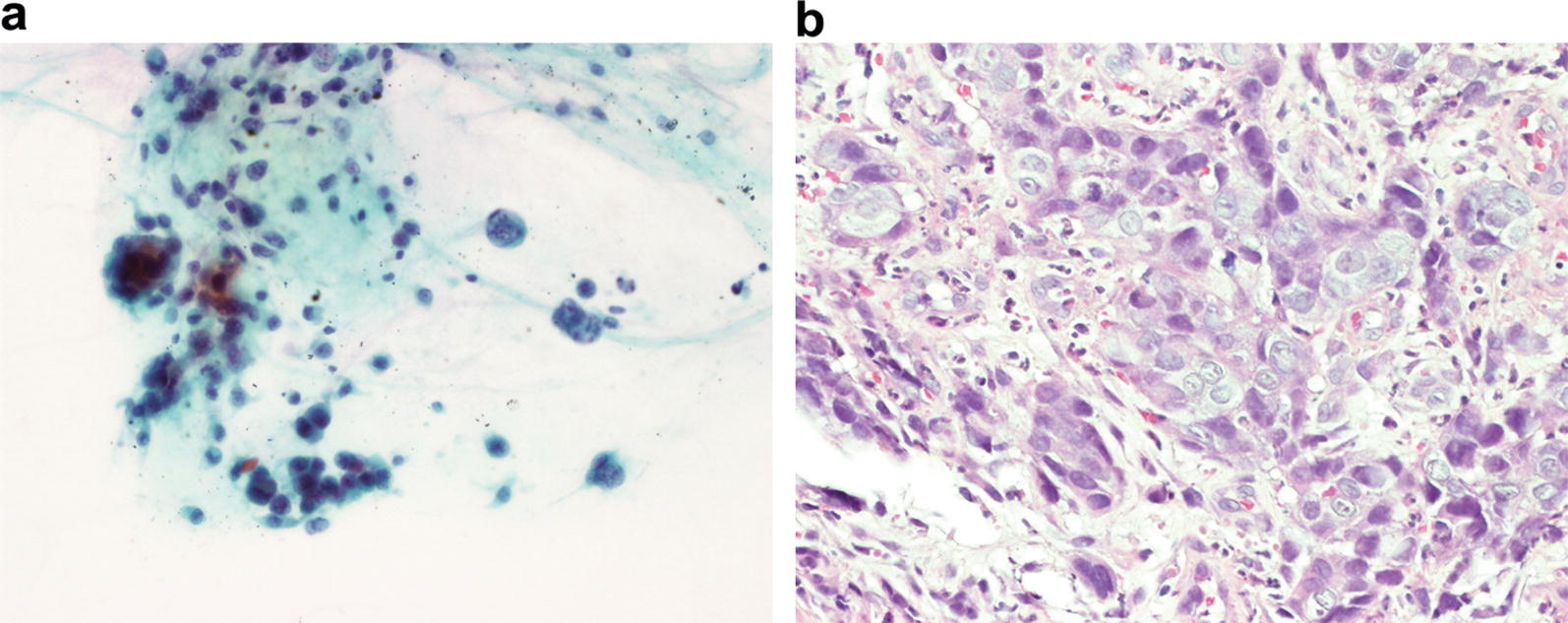
**a** Cytological analyses of a vitreous sample, stained with Papanicolaou, revealed scattered, undifferentiated, malignant cells that were consistent with the previous diagnosis of esophageal cancer. **b** Histopathological analysis from the initial esophageal biopsy, stained with hematoxylin and eosin, revealed an anaplastic, squamous neoplasm with cohesive cells. Each sample was observed with a microscope of 400 magnification

PCR testing from vitreous sample was negative for toxoplasma, cytomegalovirus, herpes simplex virus, varicella-zoster virus, bacteria, and fungi. However, the vitreous sample did contain scattered, undifferentiated malignant cells (Fig. 2a). Further immunohistochemical examination was not performed due to the small sample size. Vitreous specimen findings matched those of the primary esophageal tumor biopsy (Fig. 2b). Given the presence of a central nervous system metastasis, magnetic resonance imaging (MRI) of the head was performed. No evidence of further central nervous system malignancies was found. However repeat PET–CT revealed widespread systemic metastases.

**Fig. 3:**
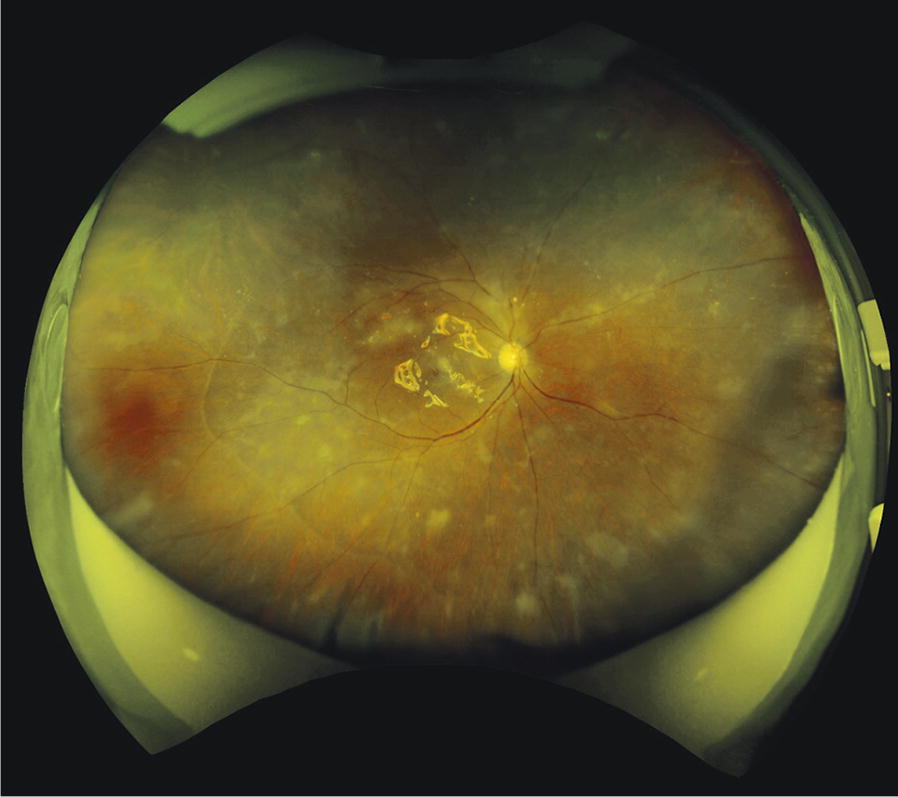
Ultra-wide view fundus imaging was obtained two months after combined cataract extraction and 25 gauge pars plana vitrectomy. Multifocal white intraretinal lesions were present in the macula and periphery. A tractional retinal detachment was resolved under silicone oil. Visual acuity remained 20/200

**Fig. 4:**
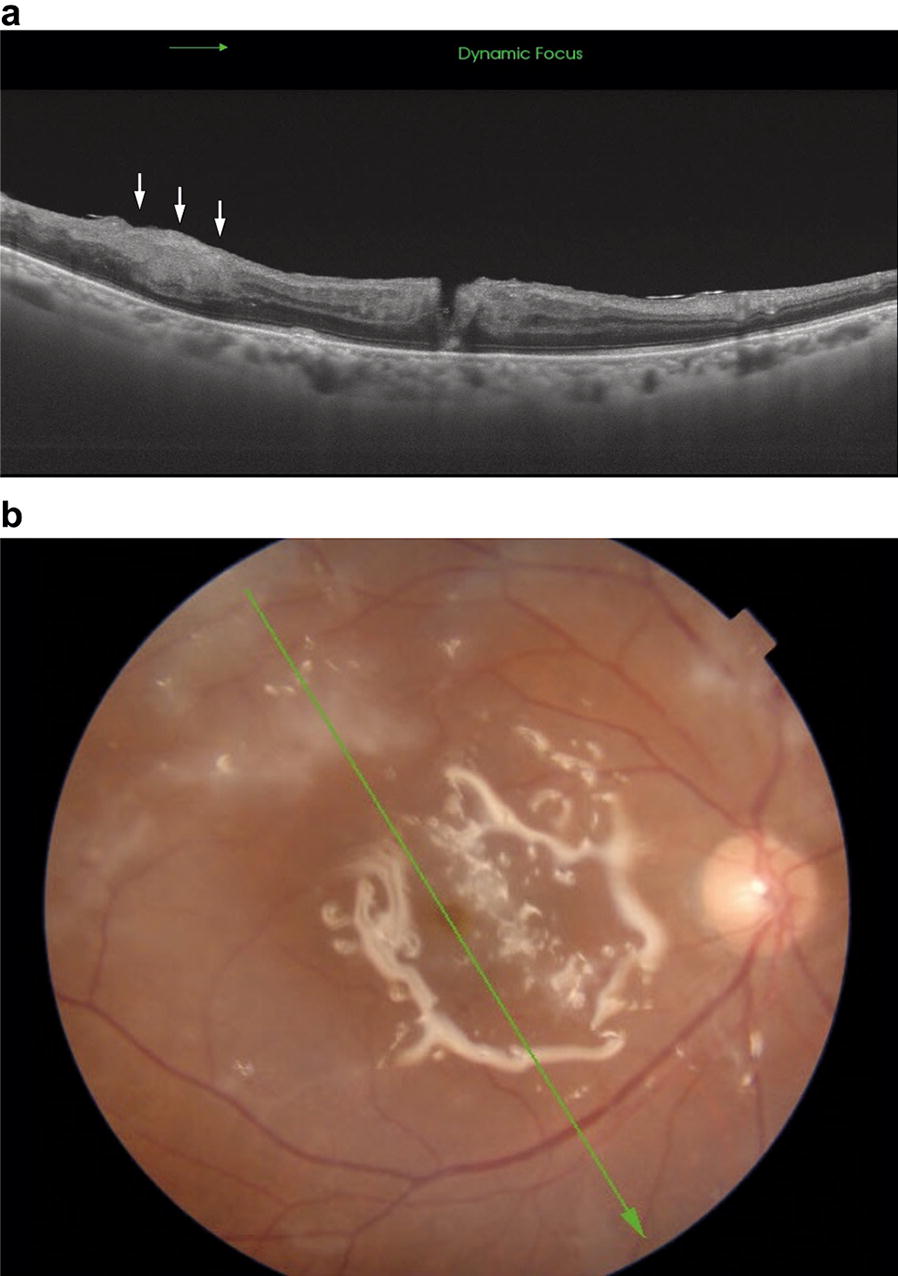
**a** OCT image showing hyper-reflective inner retinal lesions (arrows) in the same location as the white intraretinal fundus lesions. **b** Fundus photography illustrated white intraretinal lesions with a green arrow indicating the direction and area analyzed by OCT

Ultra-wide view fundus imaging revealed multifocal white intraretinal lesions in the macula and periphery two months after surgery (Fig. 3). Optical coherence tomography (OCT) through these white opacities displayed hyper-reflective inner retinal lesions with no choroidal involvement, suggestive of retinal metastasis (Fig. 4a, b). Visual acuity was 20/200 OD and the retina remained attached under the silicone oil. No further intervention was provided by our department due to the poor prognosis. The patient was maintained on palliative care and passed away three month later due to multiple organ failure, secondary to his malignancy.

## Discussion

Metastatic cancer involving the retina is a rare entity. In a 40-year retrospective study, only 8 or .3% had retinal metastasis in comparison to 2076 patients with uveal metastasis [3]. Throughout the literature, less than 35 cases of retinal metastasis without choroidal involvement have been documented. Retinal metastases in these cases were most commonly from known primary tumors such as cutaneous melanoma, breast carcinoma, gastrointestinal adenocarcinoma, and lung carcinoma.

The diagnostic difficulty of retinal metastasis lies not only in its rarity but also in its ability to mimic several pathologies. Retinal metastasis may present as whitish patches or vitreous opacities, mimicking an inflammatory, infectious retinitis, or vascular occlusive disorders. Our patient presented with a veil-like vitreous opacity, a tractional retinal detachment, and white retinal lesions throughout the macula and periphery. Our initial thought was an infectious retinitis, such as ARN. Also, the patient was immunosuppressed from chemotherapy and radiation treatment for his systemic cancer, making him more susceptible to infectious entities. However, since our patient was known to have an aggressive, stage 4 neoplasm, tumor dissemination was high on the differential as well. Thus in order to attain a definitive diagnosis and better visualize of the fundus, surgical intervention was scheduled immediately. A vitreal biopsy was performed in conjunction with cataract extraction and vitrectomy. PCR and histopathological analyses were negative for all infectious agents but confirmed a poorly differentiated adenocarcinoma identical to the original tumor biopsy.

Definitive diagnosis of retinal masses has been documented with several techniques including fine needle aspiration biopsy [8], localized retinectomy [5], and transscleral chorioretinal biopsy [9]. A retinal biopsy through any of these techniques was not performed initially as the clinical presentation was more suggestive of an infectious agent than neoplasm. Thus only vitreous samples were analyzed with the patient receiving systemic antivirals until biopsy results were obtained.

Diagnosis of retinal metastasis was supported without further biopsy by high definition imaging from OCT and ultra-widefield fundus images. Images obtained with OCT revealed hyper-reflective inner retinal lesions of the macula which simulated ARN on initial presentation (Fig. 4). However, these lesions were still present 2 months after surgery. Additionally, retinal thinning did not occur following vitrectomy, which is a known process with ARN [10]. Therefore, the hyper-reflective lesions, which tested negative for infectious entities on PCR, likely represented tumor dissemination found on vitreous biopsy. Also, ultra-widefield fundus images revealed white intraretinal lesions scattered throughout the posterior pole and periphery, without choroidal masses or lesions. The multifocal intraretinal lesions depicted in our patient may have been due to the poorly differentiated nature of the cancer, as other cases of low differentiated small cell lung carcinoma depicted similar findings [5, 11]. Lastly, ocular ultrasonography was not performed on our patient as the ultrasound would not have been sensitive enough to illustrate the flat retinal lesions seen clinically (Fig. 3).

Treatment of eyes with retinal metastasis is generally conservative [3]. Common therapy includes observation, palliative external beam radiation [5] and plaque radiotherapy [3]. More invasive and less common options include retinectomy and enucleation in cases of intractable pain. However, patient prognosis with retinal metastasis is often very poor and guarded. Life expectancy may range from 1 to 10.5 months from initial diagnosis [3, 5]. The poor outcomes documented in a majority of cases are likely due to the end-stage nature of retinal metastasis. By the time retinal dissemination has occurred from a primary tumor, overwhelming systemic metastases are usually present in the patient, leading to a high risk of mortality from systemic organ failure.

Our patient was previously diagnosed with an aggressive stage-4 esophageal carcinoma and received radiotherapy and systemic chemotherapy including three cycles of 5-FU and CDDP. However, once the diagnosis of retinal metastases was confirmed by vitreous biopsy and fundus imaging, widespread systemic metastases were present in the lung, liver, lumbar vertebrae, and mediastinal lymph nodes of our patient. MRI and PET–CT revealed no CNS lesions excluding the retina. Esophageal carcinoma can metastasize to the brain [12], thus the absence of multifocal CNS involvement outside the eye may have been due to 5-FU and its ability to cross the blood-brain-barrier (BBB) [13] yet it might not reach therapeutic levels in the retina.

## Conclusion

Retinal metastases are rare and may present as whitish intraretinal lesions or vitreous opacities, mimicking an inflammatory, infectious, or neoplastic process. Our patient presented with a veil-like vitreous opacity, a tractional retinal detachment, and white retinal lesions masquerading as an ARN. Although no retinal biopsy was performed, retinal metastasis was supported via vitreous biopsy using PCR, histopathology, OCT, and ultra-widefield fundus images. Retinal metastasis is associated with a poor prognosis and systemic tumor dissemination. Our patient received systemic chemotherapy including 5-FU which may have limited extra-ocular CNS metastasis due to its ability to cross the BBB, however, 5-FU might be inadequate in preventing vitreoretinal metastasis due to mechanisms which warrant further investigation.

## Abbreviations

ARN: acute retinal necrosis
5-FU: 5-fluorouracil
CDDP: cisplatin
OD: right eye
OS: left eye
CNS: central nervous system
PET–CT: positron emission tomography–computed tomography
PCR: polymerase chain reaction
OCT: optical coherence tomography
BBB: blood–brain-barrier
